# Expert Opinion on Pegloticase with Concomitant Immunomodulatory Therapy in the Treatment of Uncontrolled Gout to Improve Efficacy, Safety, and Durability of Response

**DOI:** 10.1007/s11926-022-01055-9

**Published:** 2022-02-15

**Authors:** John K. Botson, Herbert S. B. Baraf, Robert T. Keenan, John Albert, Karim R. Masri, Jeff Peterson, Christianne Yung, Brigid Freyne, Mona Amin, Abdul Abdellatif, Nehad Soloman, N. Lawrence Edwards, Vibeke Strand

**Affiliations:** 1 Orthopedic Physicians Alaska 3801 Lake Otis Pkwy, Anchorage, AK 99508 USA; 2 The Center for Rheumatology and Bone Research, 2730 University Blvd. West, Suite 310, Wheaton, MD 20902 USA; 3 Duke University School of Medicine Duke Medicine Circle, 124 Davison Building, Durham, NC 27710 USA; 4 Rheumatic Disease Center, 7080 N. Port Washington Road, Glendale, WI 53217 USA; 5 Rheumatology OnDemand, LLC 405 Welwyn Rd, Henrico, VA 23229 USA; 6 The Seattle Arthritis Clinic, Kirkland, WA 98033 USA; 7 Private Practice, 2482 W Horizon Ridge Parkway, Suite 130, Henderson, NV 89052 USA; 8 Rheumatology Internal Medicine 39755, Murrieta Hot Springs Rd, Ste. F110, Murrieta, CA 92563 USA; 9 Arizona Arthritis and Rheumatology Associates, 11943 East Beryl Ave, Scottsdale, AZ 85259 USA; 10 Baylor College of Medicine, 600 N Kobayashi Rd., Ste 312, Webster, TX 77598 USA; 11 Arizona Arthritis and Rheumatology Associates, 9097 W Roberta Ln, Phoenix, AZ 85383 USA; 12 University of Florida, 1600 SW Archer Road, Room 4102, Gainesville, FL 32610 USA; 13 Division of Immunology/Rheumatology, Stanford University, 306 Ramona Road, Portola Valley, CA 94028 USA

**Keywords:** Antidrug antibodies, Gout, Immunogenicity, Immunomodulation, Pegloticase

## Abstract

**Purpose of Review:**

Gout is a systemic disease from which some patients develop numerous painful tophi that adversely affect quality of life and functionality. Some patients treated with oral urate-lowering therapy are unable to maintain serum urate levels below 6 mg/dL, and these patients, thus classified as having refractory or uncontrolled gout, often require therapy with pegloticase to reduce symptoms and tophaceous burden. The objective of this expert opinion review is to summarize the available evidence supporting the use of concomitant immunomodulators with pegloticase to prevent development of anti-drug antibodies (ADAs) when treating patients with uncontrolled gout.

**Recent Findings:**

Emerging evidence suggests that adding an immunomodulator to pegloticase therapy can substantially increase response rates to double those observed in phase 3 randomized controlled trials.

**Summary:**

The combination of immunomodulation with pegloticase should be considered in routine clinical practice to improve durability of response, efficacy, and safety among patients with uncontrolled gout who otherwise have limited therapeutic options.

## Introduction

The use of biologic agents has led to important treatment advances for multiple rheumatologic conditions. Historically, biologic agents have been associated with the formation of anti-drug antibodies (ADAs), which may result in loss or lack of efficacy and/or adverse effects [1, 2•]. The co-administration of biologic agents with immunomodulating therapies that inhibit proliferation of activated lymphocytes has been shown to prevent or minimize ADA development, improve response, and lengthen therapy duration [3–6]. This approach has increased in use among clinical rheumatologists [1, 2•, 7, 8] and has shown success when treating patients with chronic refractory gout, hereafter referred to as uncontrolled gout, with the biologic agent, pegloticase [8–13, 14••, 15].

Uncontrolled gout poses a burden for patients and a challenge for treating physicians. Uncontrolled gout affects a small subset of the 9.2 million individuals with gout in the USA [16, 17]. Gout is a progressive, systemic, crystal deposition disease arising from persistent elevations of serum urate (SU) [18]. Imaging studies have shown urate crystal deposits in joints, soft tissues, cartilage, and internal organs [18–22]. Dual-energy computed tomography has demonstrated extra-articular monosodium urate (MSU) deposition, or tophi, where clinical examination may not [23–25]. Recent guidelines recommend that patients with gout maintain SU levels ≤ 6 mg/dL to eliminate crystalline deposits and resolve clinical manifestations of gout [26]. Patients with uncontrolled gout are either unable to achieve or maintain SU levels ≤ 6 mg/dL or derive adequate clinical benefit due to their extensive urate burden despite treatment with oral urate-lowering therapies [26, 27]. Extensive literature has shown that the degree of SU lowering is proportional to the speed by which urate deposits can be eliminated [28–30]. Treatment with pegloticase, the only biologic therapy currently approved in the USA for treatment of uncontrolled gout, has been demonstrated to resolve tophi and improve pain and function [28, 31–34]. The 2020 American College of Rheumatology (ACR) guidelines recommend pegloticase as third-line treatment for patients with uncontrolled gout, including those with chronic tophaceous gouty arthropathy for whom SU thresholds well below 6 mg/dL may be preferable [26].

This paper reviews the evidence supporting the use of concomitant immunomodulators with pegloticase to prevent development of ADAs when treating patients with uncontrolled gout with pegloticase. Our assessment was guided by identification of immunogenicity as the primary factor leading to loss of response in phase 3 randomized controlled trials (RCTs) of pegloticase and recent publications demonstrating improvement due to prevention of ADA formation with use of antiproliferative agents [31, 33, 35].

## Efficacy and Safety of Pegloticase

Pegloticase, a pegylated recombinant mammalian uricase, is a US Food and Drug Administration–approved medication for the treatment of uncontrolled gout [34, 36]. This enzyme catalyzes the conversion of uric acid to allantoin, a compound that is readily removed from the body through renal excretion [36]. Previously published RCTs of pegloticase in patients with uncontrolled gout intolerant to conventional urate-lowering therapies with SU levels ≥ 8.0 mg/dL reported significant tophi resolution, reduced gout flares, and improvements in tender joint counts and patient reported health-related quality of life [37, 38]. The primary end point of these RCTs was the proportion of patients who achieved SU levels < 6.0 mg/dL for ≥ 80% of the time during months 3 and 6 of active treatment versus placebo [37]. Overall, 42% who received biweekly pegloticase were responders [37].

A secondary end point was complete resolution of prespecified tophi at 6 months, which was confirmed in 45% of subjects who received pegloticase compared with 7% with placebo (*p* = 0.002) [37]. Across pooled study groups, infusion reactions (26% vs 5% of patients, respectively) were the second most common adverse event; gout flares being most common [30, 36]. Investigator-identified serious infusion reactions, none of which resulted in hospitalizations or death, occurred in 5% of subjects who received biweekly pegloticase vs none with placebo [31].

The relationship between therapeutic responses to pegloticase, development of ADAs, and risk of infusion reactions was determined in a post hoc analysis of the phase 3 RCTs [31]. In the phase 3 RCTs, ADAs were detected in 89% of patients at least once during the 6-month studies. High-titer ADAs (> 1:2430) were detected in 41% of subjects and were significantly associated with loss of therapeutic response (*p* < 0.001) [31] and subsequent risk of infusion reactions with continued infusions [31, 33, 39]. Results from these analyses indicated that monitoring for elevated pre-infusion SU levels could identify patients at risk for development of ADAs and thus infusion reactions. These recommendations are included in the prescribing information [35, 36].

## Anti-drug Antibodies and Immunomodulation

Pegloticase is an effective therapy in uncontrolled gout, yet its use is limited in a substantial proportion of patients by the development of high-titer-binding ADAs directed against the polyethylene glycol (PEG) moiety of the overall molecular structure [35, 37]. Formation of pegloticase ADAs has no direct inhibitory effect on uricase activity; instead, ADAs increase clearance of the molecule to a degree that its beneficial effects on SU levels is reduced and risk of infusion reactions increased [33, 35, 39]. Although the risk of infusion reactions due to ADAs can be substantially lowered by using the SU monitoring protocol and withdrawing treatment when SU levels increase to > 6 mg/dL [31, 33, 35, 39], a more effective approach is to abrogate formation of ADAs [1]. Abrogation by cotreatment with antiproliferative agents has been the subject of a growing body of literature. Such agents, traditionally referred to as conventional disease-modifying antirheumatic drugs (cDMARDs), include methotrexate (MTX), azathioprine (AZA), leflunomide (LEF), and mycophenolate mofetil (MMF). These cDMARDs, which reversibly inhibit purine or pyrimidine synthesis are better tolerated and more efficacious than traditional immunosuppressants [1, 3–6, 8–12, 14••].

Various case reports, proof-of-concept case studies, and ongoing RCTs have shown favorable outcomes by combining pegloticase with immunomodulation in patients with uncontrolled gout. Response rates from these studies range from 60 to 100% (Table 1), nearly double the reported 42% response with pegloticase monotherapy in phase 3 RCTs [8–13, 14••, 15, 40••].

**Table 1:** Summary of data: pegloticase and immunomodulation

Author	Type of study^a^	IMT and run-in (time frame)^b^	Responders,*n*/*N* (%)
Berhanu et al. [9]	Case report	Azathioprine (2 weeks)	1/1 (100)
Freyne [11]	Case report	Mycophenolate mofetil and cyclosporine (background therapy)	1/1 (100)
Botson et al. [14••]	Proof-of-concept case series	Methotrexate (4 weeks)	10/10 (100)
Albert et al. [8]	Case series	Methotrexate (14–35 days)^c^	8/10 (80)
Bessen et al. [10]	Case series	Methotrexate, azathioprine, or cyclosporine (at first infusion)	7/7 (100)
Masri et al. [12]	Retrospective case study	Leflunomide (variable)	4/6 (67)
Botson et al. [15]	Open-label pilot study (MIRROR)	Methotrexate (4 weeks)	11/14 (79)
Rainey et al. [13]	Open-label pilot study (TRIPLE)	Azathioprine (2 weeks)	6/10 (60)^d^
Khanna et al. [40••]	Randomized controlled trial (RECIPE)	Mycophenolate mofetil (2 weeks)	19/22 (86)

^a^Studies presented here are not head-to-head trials designed or statistically powered to compare the efficacy or safety of pegloticase alone or in combination with immunomodulation

^b^All patients received a biweekly infusion of pegloticase 8 mg in combination with the immunomodulatory therapy shown

^c^One patient received oral methotrexate 14 days after the initial pegloticase infusion

^d^Excludes 2 patients for whom treatment is ongoing

*IMT* immunomodulatory therapy

Case reports by Berhanu et al. [9] and Freyne [11] first demonstrated successful treatment with pegloticase and concomitant use of AZA and MMF/cyclosporine, respectively, in patients with uncontrolled gout. Of note, the patient described in Berhanu et al. [9] experienced 2 increases in SU levels (1.0 mg/dL and 6.2 mg/dL, respectively) attributable to AZA nonadherence, which rapidly reversed to < 1.0 mg/dL after reinstitution of AZA [9].

A proof-of-concept case series conducted by Botson and Peterson evaluated the utility of co-administration of oral MTX (15 mg weekly) with pegloticase (8 mg biweekly) in 10 patients with uncontrolled gout [14••]. All 10 patients achieved a full therapeutic response and reached the primary end point, defined as the proportion of patients who maintained SU levels < 6.0 mg/dL for 80% of the time between months 3 and 6. In addition, all patients completed full courses of treatment without increases in SU levels or infusion reactions [14••].

Albert et al. [8] conducted a case series in 10 patients with uncontrolled gout where administration of oral or subcutaneous MTX with pegloticase led to an 80% response rate. Nine of 10 patients received weekly injections of subcutaneous MTX (25 mg); 1 patient 12.5 mg orally weekly. Two patients discontinued therapy before receiving the full 12-week infusion course: 1 due to loss of response and a mild infusion reaction and 1 to personal preference [8].

Bessen et al. [10] reported 7 patients with uncontrolled gout treated with MTX, AZA, or the immunosuppressive cyclosporine with pegloticase, initiated on the first day of infusions. Six of 7 received MTX; 1 patient was switched to azathioprine at the fifth pegloticase infusion due to fatigue related to MTX. Cyclosporine (50 mg twice daily [BID]) was chosen for 1 other patient due to elevated liver enzymes at baseline. All patients maintained SU levels ≤ 6 mg/dL (100% response), and no infusion reactions were reported [10].

To assess responses in patients who received LEF co-administration with pegloticase, Masri et al. [12] retrospectively studied 10 with uncontrolled gout. Of 6 patients analyzed at the time of data cutoff, 4 met the primary end point (defined as those who received ≥ 12 pegloticase infusions), and 2 were lost to follow-up, yielding a 67% overall response rate [12].

RECIPE (Reducing Immunogenicity to Pegloticase), an investigator-initiated proof-of-concept RCT, examined whether a short course of MMF could effectively and safely reduce immunogenicity to pegloticase [40••]. Patients with uncontrolled gout were randomized 3:1 to receive either 1 g MMF BID (*n* = 22) or placebo (*n* = 10), with a 2-week run-in prior to initiating pegloticase biweekly. After 12 weeks, patients in the MMF arm had MMF withdrawn and both arms were followed for another 12 weeks. Eighty-six percent (19 of 22) in the MMF arm achieved the primary endpoint of SU ≤ 6 mg/dL at 12 weeks, compared with 40% (4 of 10) in placebo. At 24 weeks, serum uric acid response was sustained in 68% of the patients in the MMF arm, whereas the response rate for the placebo group further decreased to 30%. The proportion of patients with sustained SU levels < 6 mg/dL at both 12 and 24 weeks was significantly higher with MMF in both groups (*p* = 0.02 and *p* = 0.03, respectively). Furthermore, there were no infusion reactions in the MMF group compared with 30% in patients receiving placebo [40••].

MIRROR-OL (Methotrexate to Increase Response Rates in Patients with Uncontrolled Gout Receiving Pegloticase), an open-label pilot study in adult patients with uncontrolled gout (*N* = 14), further evaluated the use of MTX with pegloticase [15]. The primary end point was the proportion of responders, defined as those who maintained SU levels < 6 mg/dL ≥ 80% of the time during month 6 of pegloticase treatment [15]. Preliminary results showed a response rate of 78.6% (*n* = 11/14), based on a modified intention-to-treat population, defined as those who received ≥ 1 pegloticase infusion. One investigator-reported infusion reaction, identified as a mild cough, occurred in a patient during their fifth pegloticase infusion; however, this patient still completed 24 weeks of treatment as a responder. Responders at 6 months who continued treatment (*n* = 8) remained responders at month 12 [15].

TRIPLE (Tolerization Reduces Intolerance to Pegloticase and Prolongs the Urate Lowering Effect) included patients with uncontrolled gout cotreated with AZA and pegloticase (*N* = 12); results were reported in an interim analysis. Patients received 1.25 mg/kg AZA daily for 1 week, increased to 2.5 mg/kg before the first pegloticase infusion, followed by 2.5 mg/kg daily for the trial duration [13]. To date, 6 patients have completed the treatment course, and 2 remain on treatment, all with persistent urate-lowering effects resulting in levels below the predefined SU threshold (< 6 mg/dL). Of the remaining 4 patients not included in the analysis, 2 lost urate-lowering efficacy, 1 experienced an infusion reaction during the first dose, and 1 had subjective intolerance to AZA [13].

PROTECT (Prospective Study of Pegloticase in Transplant Patients), a phase 4, multicenter, open-label study, evaluated the efficacy of pegloticase in adults with uncontrolled gout who underwent kidney transplant and were receiving post transplantation immunosuppressant therapy [41•]. Results from the completed trial demonstrated an 88.9% response rate (16/18 patients), with 2 patients removed due to COVID infection/COVID concerns [42].

Finally, MIRROR-RCT (Methotrexate to Increase Response Rates in Patients with Uncontrolled Gout Receiving Pegloticase), a multicenter, randomized, double-blind, placebo-controlled study in adult patients with uncontrolled gout showed a 71% response rate in the methotrexate-treated group vs. 38.5% in the placebo group with the primary endpoint defined as the proportion of responders who maintained SU levels < 6 mg/dL at least 80% of the time during month 6 [43].

**Fig. 1:**
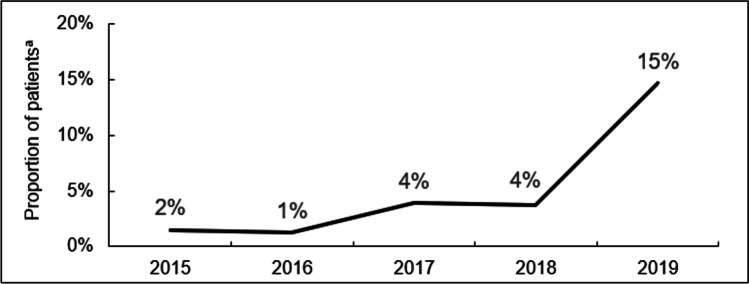
Proportion of patients receiving pegloticase and concomitant immunomodulatory therapy by year (2015–2019) [45•]. ^a^Patients who started either methotrexate or azathioprine within 60 days of their first pegloticase infusion

A summation of a number of the previously described cases was included in a systematic review of 10 publications (describing 82 cases) using immunomodulation in the setting of pegloticase administration that demonstrated a marked improvement in responder rates (to 82.9% overall), compared with pegloticase monotherapy. Of the 80 cases on a single immunomodulatory agent, response rates of 90.3% for oral MTX, 86.4% for MMF, 77.8% for subcutaneous MTX, 66.7% LEF, and 63.6% AZA [44]. Although it should be noted the case numbers are small and not designed to make comparisons between agents. As pegloticase is the only medication indicated for treatment of uncontrolled gout and often implemented as a last resort, maximizing the efficacy of such therapy is of paramount importance. As reviewed, practicing physicians, including authors of this publication, have opted to initiate immunomodulatory therapy before or in conjunction with pegloticase therapy to attenuate ADA formation and increase the potential for more durable responses. A retrospective claims analysis detailing the proportion of patients receiving concomitant immunomodulatory therapy with pegloticase by year (2015–2019) is shown in Fig. 1 [45•] with a dramatic increase observed following an ACR 2018 presentation detailing use of antiproliferative immunomodulators with pegloticase [45•].

### Considerations

Patients with uncontrolled gout often have numerous comorbidities, including renal dysfunction, cardiovascular disease and a history of alcohol use or liver disease, and rheumatologists considering the use of an immunomodulator with pegloticase have several agents from which to choose [18, 46–49]. The availability of multiple antiproliferative therapies provides the flexibility to tailor therapy to the specific patient, crucial in those with multiple comorbidities requiring dose adjustments, hematologic testing, and/or other risk management measures (Table 2) [47–50]. Although the addition of these immunomodulators may increase risk for infection, their use is only temporary during active treatment and studies thus far have not shown an increased infection rate [47–50]. Data from the pending MIRROR-RCT will provide greater clarity [51••]. Given the progressive, often erosive nature of tophaceous gout and limited treatment options, it is critical to consider implementation of one of these immunomodulatory agents in conjunction with pegloticase to maximize the likelihood of a full and durable response to therapy [18].

**Table 2:** Immunomodulator considerations for use

Immunomodulator	Considerations		
	Hepatic	Renal	Other
Methotrexate [48]	• Avoid in preexisting liver disease • Toxic interaction with alcohol • Monitor CBCs and LFTs	• Dose adjustment for renal impairment	• Contraindicated with pregnancy
Leflunomide [49]	• Contraindicated with hepatic impairment • Liver function monitoring	• Patients with a history of renal failure or transplant may already be taking this medication	• Contraindicated with pregnancy
Mycophenolate mofetil [50]	• Dose adjustment for adult transplant patients • Monitor CBCs and LFTs	• Patients with a history of renal transplant or lupus nephritis may already be taking this medication	• Potential for severe and limiting GI, glucose, and cholesterol side effects • Contraindicated with pregnancy
Azathioprine [47]	• Potentially hepatotoxic in transplant patients • Monitor CBCs and LFTs	• Dose adjustment for renal impairment	• Drug–drug interaction with allopurinol • **Prescreen for TPMT**

*CBC* complete blood cell count; GI, gastrointestinal, *LFT* liver function test, *TPMT* thiopurine S-methyltransferase

## Conclusions

Based on the review of an accumulating body of evidence, concomitant immunomodulatory therapy administered with pegloticase can significantly improve durability of response, efficacy, and safety among patients with uncontrolled gout, for whom this may be the only treatment option. Current data from observational case studies, MIRROR-OL and RECIPE, demonstrate the consistency of these responses. Given the widespread use of antiproliferative agents to abrogate immunogenicity with other biologic agents for the treatment of rheumatic diseases, clinical rheumatologists should feel comfortable with similar treatment regimens with use of pegloticase for treatment of uncontrolled gout.
